# Correlation between Maspin Levels in Different Biological Samples and Pathologic Features in Colorectal Adenocarcinomas

**DOI:** 10.3390/life13041060

**Published:** 2023-04-20

**Authors:** Alexandru Adrian Bratei, Raluca-Ioana Stefan-van Staden

**Affiliations:** 1 Faculty of Chemical Engineering and Biotechnologies, University Politehnica of Bucharest, 060042 Bucharest, Romania; 2 Laboratory of Electrochemistry and PATLAB, National Institute of Research for Electrochemistry and Condensed Matter, 060021 Bucharest, Romania; 3 Department of Pathology, Emergency University Hospital, 050098 Bucharest, Romania

**Keywords:** maspin, colorectal adenocarcinoma, stochastic microsensors, biomedical analysis

## Abstract

Maspin is an important biomarker which was proven to be correlated to many pathological features that can help the oncologists, the surgeons and also the pathologists for choosing the personalized treatment of the patients. Maspin expression correlates with the budding of colorectal adenocarcinomas that is usually used mostly in immunohistochemistry. In this preliminary study, a small number of patients with clinical and pathological features were selected. Four kinds of samples (tumoral tissues, blood, saliva and urine) were analyzed using a stochastic method using stochastic microsensors. Whole blood maspin concentration values were related to budding, molecular subtype and location. Tissular maspin concentrations were related to location, maxi-mum diameter and pN value from TNM staging system. Salivary maspin concentrations were related to budding, mucinous compound and macroscopic features. Urinary maspin concentrations were related to pT value from TNM staging system, budding and molecular subtype. The correlations made in this paper may be used for fast diagnostic of colorectal adenocarcinomas, after which, it will be tested on a significant number of patients confirmed with colon cancer, in different stages of evolution.

## 1. Introduction

Maspin is a protease inhibitor of the serpin family, known to have tumor suppressor activity. First identified in 1994 [1], maspin was called a multifaced protein, because it interacts with diverse groups of intercellular and extracellular proteins, and regulates cell adhesion, motility, apoptosis, and angiogenesis [2]. Some human cancers, such as breast carcinoma and squamous cell carcinoma, had a prognostic related to maspin expression [3]. The loss of the maspin gene expression is correlated with an increasing invasiveness and the spreading of metastatic cells in vivo and in vitro [4]. Recently, some progress was recorded in proving that maspin can be used as biomarker for diagnosis and prognosis of tumors; it can also suggest tumor suppression and there are some cancer interventions which are maspin-based [5]. A very important pathological feature is the inverse correlation of microvessel density and p53 which indicates a regulatory effect on maspin and also the anti-angiogenesis effect associated with maspin [5]. Maspin also proved to be a prognostic marker for early stage colorectal cancer with microsatellite instability [6], as well as a biomarker for early recurrence in primary stage III and IV of colorectal cancer [7]. Umekita found that expression of maspin may be correlated with the aggressiveness of colorectal adenocarcinomas; its expression tends to decrease during the development of colon cancer from adenoma of early colon cancer, adenocarcinoma and then to metastatic tumor [8]. Its quantification using reliable methods is essential [7,8]. Studies dedicated to deciphering the metabolic mechanism indicated that the nuclear localization of maspin in cancer cells is essential for its tumor suppression activity and that if maspin is localized in the nucleus, it binds to chromatin to prevent cell division. Regarding the intracellular and extranuclear distribution, maspin is predominantly cytoplasmic, but can also be located in other cellular compartments from where it is subsequently secreted. The secreted maspin binds to the components of the extracellular matrix. A plausible hypothesis is that maspin exerts its role only in the nucleus at the level of gene or chromatin regulation and in this way, it indirectly affects the interaction between the cell and the matrix, being released as a consequence of cell damage/necrosis [9,10,11,12].

This paper proposed, based on the determination of the concentration of maspin in whole blood, saliva, urine, and tumoral tissue using reliable stochastic sensors [13], to correlate these concentrations with location, maximum diameter, and pN values from TNM staging system (using tissular maspin), budding, mucinous compound, and macroscopic features (using salivary maspin), and pT values from TNM staging system, budding, and molecular subtype (using urinary maspin). These correlations may have, as a feature, a fast diagnostic of colon cancer, with the possibility of a personalized treatment based on the determination of maspin concentration using a cost-effective method using stochastic microsensors.

## 2. Patients and Methods

### 2.1. Patient Description

A total of 31 patients confirmed with colorectal cancer, selected from the database of the project GRAPHSENSGASTROINTES, were considered for this study. The including criterion is based on a random selection of patients by the date of when the surgical intervention occurred; thus, patients which came over a span of 2 months were chosen. Informed consent was received from each of the patients before collecting the biological samples. The following samples were collected, screened, and used in the study: 31 whole blood samples, 30 tumor samples, 23 urine samples, and 22 saliva samples. All the samples were collected immediately after the diagnosis with colon cancer and before any type of treatment was provided. Protocol used for the collection of the samples was in accordance with with the Ethics Committee’s approval, nr. 32647/2018, awarded by the County Emergency Hospital from Targu-Mures. The parameters and patients’ pathological features were chosen from the database of County Emergency Hospital from Targu-Mures, where the pathologists and geneticists evaluated each case and completed the database. The access to these data was granted by the project number PN-III-P4-ID-PCCF-2016-0006 within PNCDI III.

### 2.2. Materials and Reagents

All chemicals used were of analytical grade. Maspin extract was purchased from Sigma Aldrich (St. Louis, MO, USA) and paraffin oil (d420, 0.86 g/cm^3^) from Fluka. The stochastic sensors were designed and characterized as described earlier [13].

### 2.3. Device Used in Research

All the measurements were performed using an Autolab PGSTAT 302 (Metrohm, Herisau, Switzerland) connected to a computer equipped with the GPES software. The electrochemical cell included the stochastic microsensor, the reference electrode (Ag/AgCl) and the auxiliary electrode (Pt). The construction of the stochastic microsensor comprised the following steps [13]: the NS co-doped graphene powder was mixed with paraffin oil to form a homogeneous paste; this paste was mixed with a solution of maltodextrine (1 × 10^−3^ mol L^−1^)—to 100 mg paste were added 100 μL from the maltodextrin solution—to form the modified paste; each modified paste was placed in non-conducting plastic tubes with inner diameter of 150 μm, and a length of 5 mm; silver wire served as contact between the paste and the external circuit. The stochastic microsensors were washed with deionized water and dried between measurements. When not in use, they were kept in a dry place.

### 2.4. Stochastic Method

The stochastic method was used for identification and quantification of maspin in the biological samples.

**Scheme 1:**
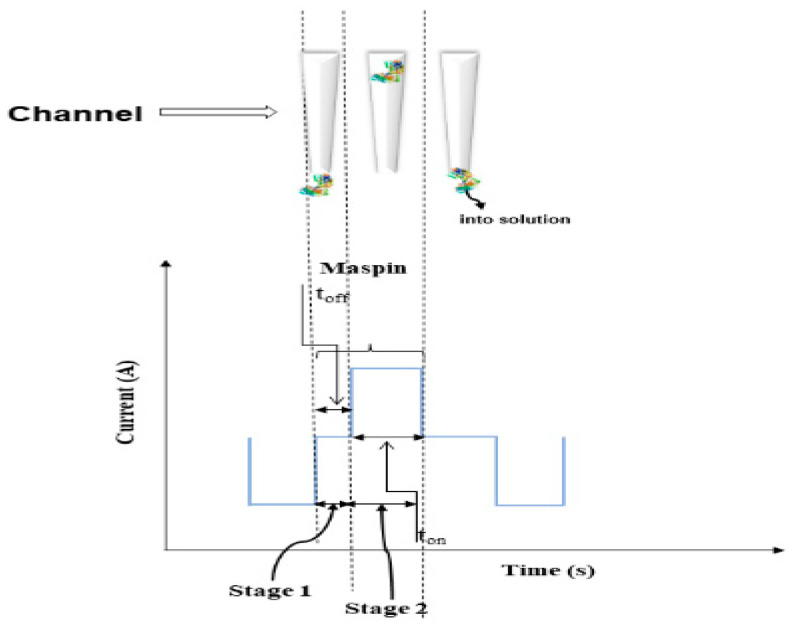
Principle used for the determination of maspin using the stochastic microsensor based on maltodextrin.

The current development in stochastic sensors is based on channel conductivity: when the biomarker is entering the channel, the current value is dropping to zero value until the molecule in inside the channel—the time needed for the molecule to enter the channel is known as t_off_ (the signature of the biomaker); this step is followed by the quantification step when the molecule (inside the channel) is taking part to binding and redox processes—this step is characterized by the t_on_ value, and this value can be found in the diagrams in between two consecutive t_off_ values (signatures). The choice of the stochastic microsensors was based on the following reasons: they can identify maspin in any biological sample, despite its complexity, because the recognition of maspin is based on its signature (t_off_ value), which depends only on the characteristics of the channel used for the sensor design, and it is not dependent of the complexity/composition of the biological sample from where it is determined (Scheme 1).

In addition, the stochastic microsensor can give valuable quantitative information about the concentration of maspin, this being correlated with the value of 1/t_on_ (Scheme 1). Stochastic sensors and microsensors used to date for biomedical analysis proved high reliability when used for assay of biomarkers in different biological samples [13,14,15,16,17].

The measurements of the maspin solutions and the biological samples were performed connecting a personal computer which had the GPES software to an AUTOLAB/PGSTAT 302 N (Methrom). All measurements were carried out at 25 °C. The biological samples were used as collected from the patients; no processing of samples was performed. A chronoamperometric technique was used for the measurements of t_on_ and t_off_ (signature of maspin) values, at a constant potential (125 mV vs. Ag/AgCl). The signature of maspin served for its identification in the diagrams obtained after the screening of the biological samples. Due to differences of pH between the urine samples and whole blood, saliva, and tumoral tissue, the calibration of the stochastic microsensors was carried out at two pH values: 3.00 (for urine samples) and 7.40 (for whole blood, saliva, and tumoral tissue).

The stochastic microsensors, the reference electrode Ag/AgCl, and the Pt wire were immersed into the biological samples. After a measurement of 6 min, a diagram was obtained. The signatures of maspin were: 3.6 s when the measurements were carried out at pH7.4, and of 3.4 s when the measurements were carried out at pH 3.00. After the identification of maspin in the diagram, in between the two t_off_ values, the t_on_ value (needed for the quantification of maspin was read). The calibration equations for maspin were obtained using the linear regression method. The unknown concentration of maspin was determined using these equations of calibration [13]:

1/t_on_ = 0.03 + 2.95 × 10^4^ × C at pH = 7.40

1/t_on_ = 0.03 + 9.68 × 10^2^ × C at pH = 3.00

At pH = 7.40, the working concentration range of the microsensor was between 41 fg mL^−1^ and 2 µg mL^−1^, with a limit of quantification of 41 fg mL^−1^.

At pH = 3.00, the working concentration range of the microsensor was between 1 pg mL^−1^ and 0.8 µg mL^−1^, with a limit of quantification of 1 pg mL^−1^.

## 3. Results

The results obtained for the concentrations of maspin in whole blood, tissue, saliva, and urine, using the stochastic sensors are given in Table S1. Additionally, the patients features used for data correlations are shown in Tables S2a and S2b.

### 3.1. Correlation of the Concentration of Maspin in Whole Blood with the Pathological Features

Correlations to budding, molecular subtype, and location were observed, as described below. For budding, patients were classified by the value of the concentration of maspin in: patients with a concentration of maspin in whole blood smaller than 160 pg/mL and patients with a concentration of maspin in whole blood higher than 160 pg/mL (Table 1).

It was observed that most patients with a whole blood concentration of maspin lower than 160 pg/mL had a budding of two or three (85.71%), and so, a smaller value can be correlated with a bigger budding value. On the other side, seven of nine (77.77%) of the patients with budding of zero and one had a value of the concentration of maspin higher than 160 pg/mL. For a concentration of maspin value higher than 160 pg/mL, the only assumption can be that it was correlated to budding one and two (11 of 15 patients).

Regarding the molecular subtype, it can be observed (Table 2) that hybrid and mesenchimal types were all associated with values of maspin concentration higher than 100 pg/mL.

Regarding the location of tumor, some aspects were also observed (Table 3). A first observation was that the highest average value was observed for ascending colon adenocarcinomas (261.51 pg/mL); in this case, it was observed that five of six patients (83.33%) had a maspin concentration higher than 180 pg/mL. Further to this, it was observed that most values for ascending and transverse colon (7 of 9—77.77%) were associated with values of maspin concentration higher than 180 pg/mL. This was reversed in the case of left half tumor where 13 of 22 patients (59.09%) were associated with values of concentration of maspin lower than 180 pg/mL. All of the segments in part had most cases with values of the concentration of maspin lower 180 pg/mL. 

### 3.2. Correlation of the Concentration of Maspin in Tumoral Tissue with the Pathological Features

A correlation between the tumor location and the concentration of maspin in tumoral tissue values was observed (Table 4). The reference concentration of maspin in tumoral tissue was 295 pg/mL. Ascending and transverse colon tumors were associated with concentrations of maspin in tumoral tissue higher than 295 pg/mL (7 of 9 patients—77.77%), while tumors found in the descending colon, sigmoid colon, rectosigmoid colon, and rectal tumors were associated with values of the maspin concentration in tumoral tissue lower than 295 pg/mL (18 of 21 patients—85.71%). This classification by the value of the maspin concentration found in the tumoral tissue can be applied to each segment in part.

**Figure 1:**
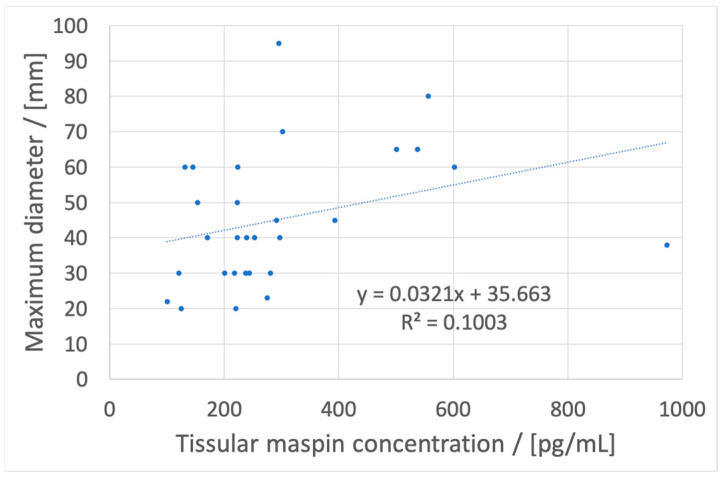
Correlation between the concentration of maspin in tumor (tissular maspin concentration) and the maximum diameter of the tumor when all values were considered.

**Figure 2:**
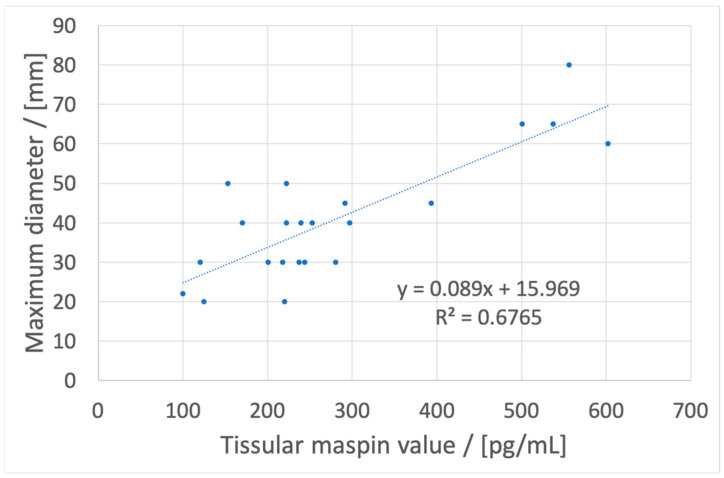
Correlation between the concentration of maspin in tumor (tissular maspin concentration) and the maximum diameter of the tumor when 22 closed values were considered.

Another correlation was with maximum diameter of the tumor. By considering all 29 values, a slightly increasing of the tumoral tissue maspin concentration with the diameter of the tumor was observed (Figure 1), but some values were distant from the trendline, and so, by eliminating them (7 of the 29 values, 24.13%), the resulting trendline seemed to have a good linear correlation to the maximum diameter of the tumor (Figure 2).

**Figure 3:**
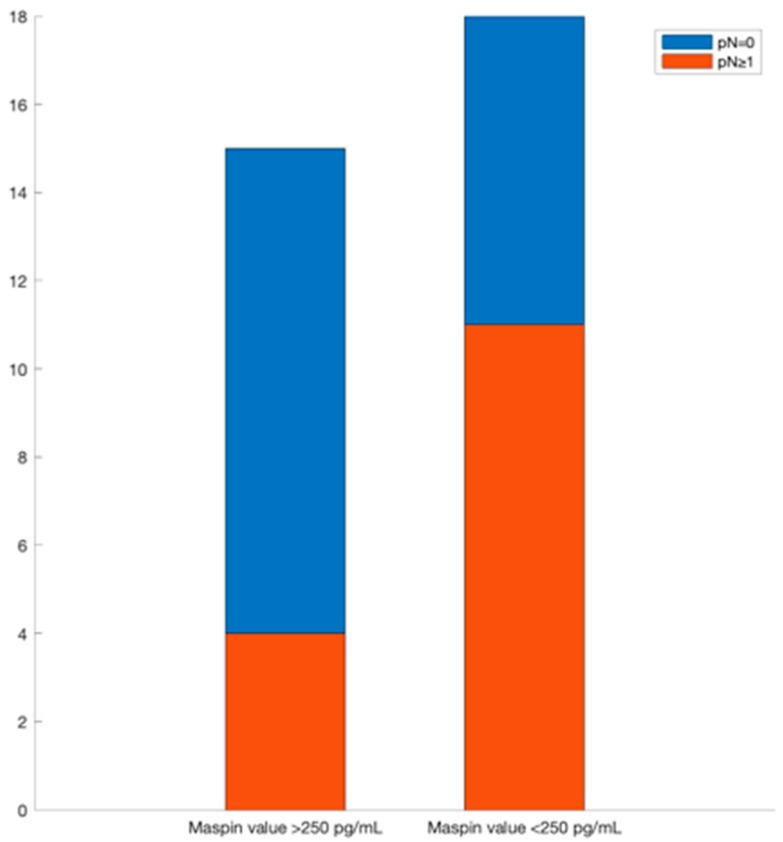
Correlation between the concentration of maspin in tumoral tissues and lymph node metastases.

The tissular values were also correlated with lymph node metastases (pN). Among the ones with values under 250 pg/mL, 11 out of 18 had lymph node metastases, while among the ones with values over 250 pg/mL, only 4 out of 15 had lymph node metastases (Figure 3).

### 3.3. Correlation of the Concentration of Maspin in Saliva with the Pathological Features

**Figure 4:**
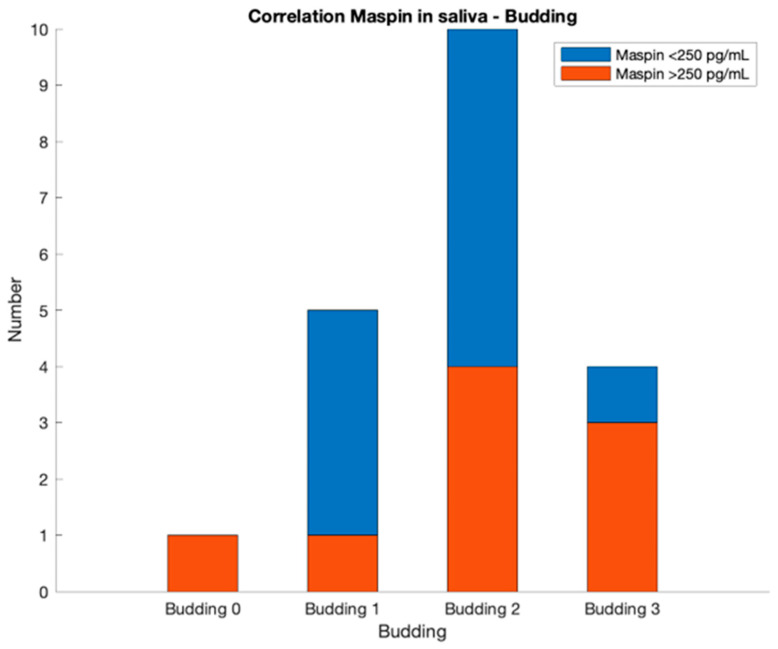
Dependence of budding value on the maspin concentration.

The values of maspin concentration in saliva were associated with budding. A higher maspin concentration value was associated with a higher budding value (Table 5). For values over 250 pg/mL, 75% of the patients had a budding of three, while only 25% had values under 250 pg/mL. For the budding value of one, 80% of them had under 250 pg/mL and only 20% of them had a maspin concentration over 250 pg/mL (Table 5 and Figure 4).

It was also observed that all the patients presenting mucinous compound had a salivary maspin concentration higher than 200 pg/mL.

A final correlation was observed between the concentration of salivary maspin and the macroscopic features, classified as vegetant and non-vegetant. The patients were classified and taken into account as reference value for the salivary maspin concentration a value of 200 pg/mL (Table 6). It was observed that five of seven patients (71.42%) with vegetant feature tumors had a value of salivary maspin concentration higher than 200 pg/mL.

### 3.4. Correlation of the Concentration of Maspin in Urine with the Pathological Features

**Figure 5:**
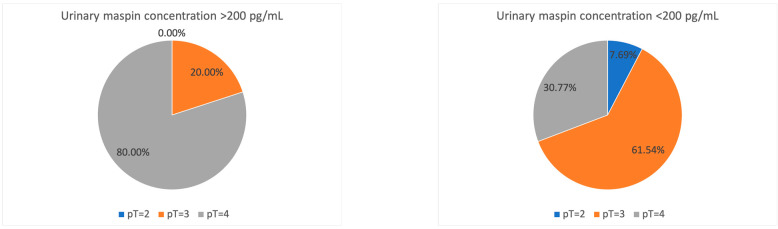
Dependence of pT values on the concentration of urinary maspin.

The values recorded for the concentration of maspin in urine were correlated with the local invasion parameter (pT) from TNM staging system. A positive correlation was observed, so that among the patients with pT = 4, 10 out of 12 patients (83.33%) had values of urinary maspin higher than 100 pg/mL and all had the concentration of urinary maspin higher than 20 pg/mL, while among the ones with pT = 3, only 2 out of 11 patients (18.18%) had values of the concentration for urinary maspin higher than 100 pg/mL and the others had the concentration for urinary maspin between 10 and 100 pg/mL (Figure 5).

**Figure 6:**
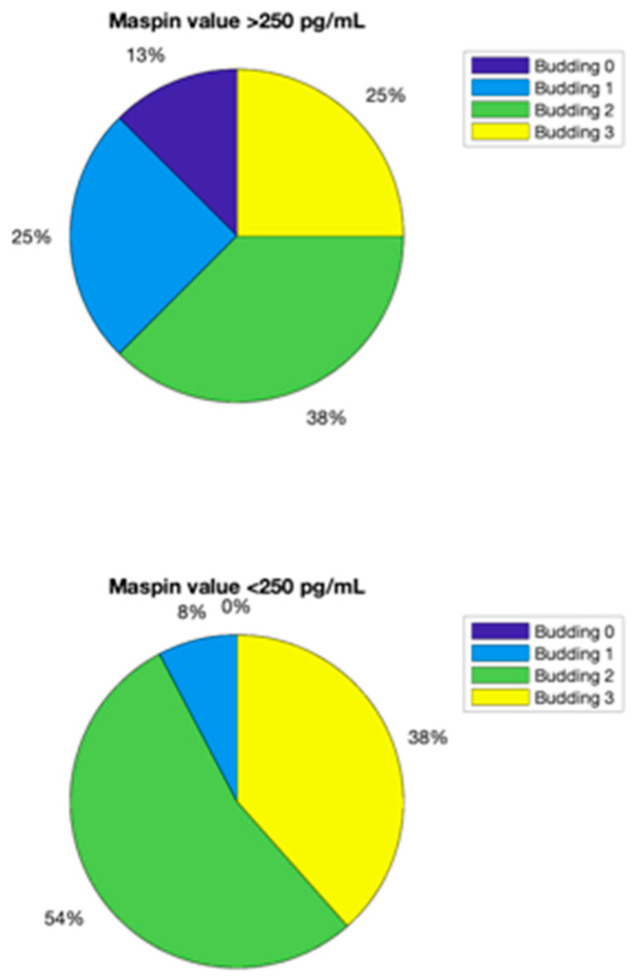
Dependence of the budding values on the urinary maspin values.

The concentrations of urinary maspin were also correlated with the budding. A negative correlation was observed, as shown in the table below, where the percents of each value of budding were calculated (Table 7 and Figure 6).

**Figure 7:**
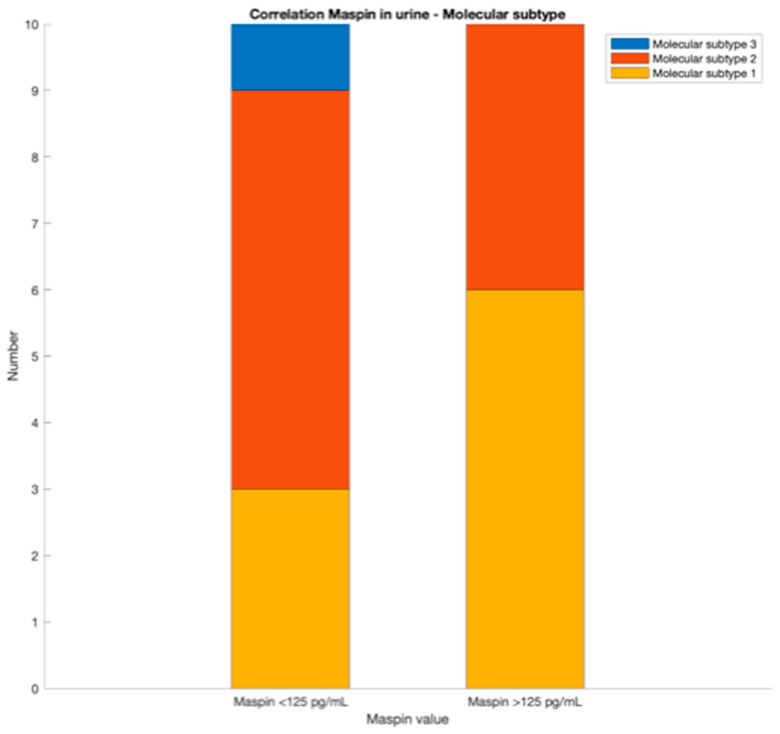
Dependence of the molecular subtype on the urinary maspin concentrations.

Another correlation was observed between the concentration of urinary maspin and the molecular subtype. A smaller value for maspin urinary concentration was associated mostly with an epithelial compound while an increment was associated with a hybrid to mesenchymal compound (Table 8 and Figure 7).

## 4. Discussion

It was recently established that maspin is epigenetically regulated at the tissue level with variations depending on each histological type [9,10,11,12]. Epigenetic changes in maspin expression involve: cytosine (de)methylation; deacetylation of histones; decreased chromatin accessibility, causing loss of gene function. In this sense, it was shown that: overexpression of maspin in gastric, pancreatic, and ovarian cancers results from demethylation of the CpG promoter (CpG promoter refers to a promoter region of a gene that contains a high frequency of CpG sites, while CpG islands are regions of DNA where a cytosine nucleotide is followed by a guanine nucleotide in the linear sequence of bases along its 5′ → 3′ direction); both methylation and demethylation of the maspin promoter could regulate the expression of the maspin gene and could guide the interpretation of up/down-regulation associated with a negative prognosis. Maspin gene promoter methylation is involved in several cancers such as breast, thyroid, skin, and colon cancers and it was admitted that this is one of the most common mechanisms that causes loss of gene function. It is noteworthy that in somatic tissues, most CpG regions are methylated and tumor cells show global hypomethylation of DNA compared to their normal counterparts [9,10,11,12]. Hypomethylation is involved in the progression from premalignant to malignant and leads to the activation of genes involved in the development of cancer [9,10,11,12].

The Kaplan–Meier analysis did not demonstrate any significant relationship between maspin expression and survival time of patients with adenocarcinoma (*p* > 0.05). Low maspin expression is associated with liver metastases of colorectal adenocarcinoma (CRA) possibly by degradation of the extracellular matrix (tenascein) to increase the mobility of adenocarcinoma-related cells [9,10,11,12]. Another study was conducted in Japan, where there was a significant increase in the incidence of colorectal cancer. Pathological and genetic research showed that colorectal adenoma is a precursor to most colorectal adenocarcinomas and that it can suffer from malignant degeneration in adenocarcinoma if not treated. However, at present, the biomolecular mechanisms underlying colorectal carcinogenesis and CRA progression are still incompletely elucidated [9,10,11,12]. The genetic study showed that the Maspin gene is associated with the human chromosome 18q21.3-q23 (in the same region as the plasminogen-2 activator inhibitor gene (PAI-2), the DCC (colorectal cancer suppressor) tumor suppressor gene and the BCL-2 gene), whose cDNA consists of 2584 nucleotides and encodes a 42 kDa polypeptide [9,10,11,12].

Maspin has significant homology to the family of serine protease inhibitors such as plasminogen activation inhibitors 1 and 2 (PAI-1 and PAI-2) and α1-antitrypsin, which are responsible for extracellular matrix degradation (ECM) [9,10,11,12]. Maspin was shown to inhibit the motility and invasion of tumor cells in in vitro breast/prostate cancer cell lines and it reduces the capacity for tumor development and metastasis of cancer cell lines in animal models [9,10,11,12]. Maspin was shown to suppress angiogenesis, induce cell apoptosis, and promote cell adhesion to the basement membrane and the ECM (extracellular matrix). Although loss of maspin expression was reported to correlate with increased aggression in advanced breast and prostate cancers, classifying maspin as a potent tumor suppressor, some in vivo studies characterized it as an important oncogene, as its overexpression was reported in cases of cancer of: pancreas, ovary, thyroid, lungs, and bladder [9,10,11,12]. Moreover, overexpression was also correlated with lymph node metastases of gastric cancer and poor prognosis of lung cancer.

It was established that maspin has different functions depending on: tissue histological type, stage, and intracellular localization. It was also established that protein interactions may be the cause of controversial results. The biological function of serpin B5 was assigned to the central reactive loop (RCL) and is involved in protein interactions and cell cohesion. In addition, there are some components of the extracellular matrix (glutathione S-transferase and interferon regulatory factor) that may interact with serpin B5 to perform its biological functions.

If a metastasis from a colon tumor is suspected, the whole blood maspin concentration can be used. If its value is under 180 pg/mL, the location is probably in the left colon and a colonoscopy can be used for obtaining a biopsy and further tissular results which will give data about the maximum diameter of the tumor and invasions in lymphatic nodes.

Another important parameter is the budding value. It can be obtained primarily from blood, urine and saliva analyses. For example, a value of 2 for budding is given by the next two combinations:

- Whole blood maspin concentration lower than 160 pg/mL, urinary maspin concentration lower than 250 pg/mL and salivary maspin concentration between 150 and 250 pg/mL;
- Whole blood maspin concentration lower than 160 pg/mL, urinary maspin concentration higher than 250 pg/mL and salivary maspin concentration lower than 300 pg/mL.

For the molecular subtype, blood and urine maspin values can be primarily used. A value of whole blood maspin concentration lower than 100 pg/mL indicates an epithelial subtype. If the value of whole blood maspin concentration is higher than 100 pg/mL and urinary maspin concentration value is lower than 125 pg/mL, a mesenchymal compound is probably implied. For a whole blood maspin concentration value higher than 100 pg/mL and a urinary maspin concentration value higher than 125 pg/mL, the tissular maspin concentration value from biopsy is necessary. If the tissular maspin concentration value is lower than 240 pg/mL, a mesenchymal compound is probably implied; also, the molecular subtype is probably an epithelial one.

For TNM staging, a urinary maspin concentration value lower than 200 pg/mL suggest a pT value of 2 or 3, while a higher concentration of urinary maspin than 200 pg/mL suggest a pT = 4; for gross features, the salivary maspin concentration value higher than 200 pg/mL increase the probability of a vegetant tumor, while a salivary maspin concentration value lower than 200 pg/mL can exclude a mucinous compound.

**Figure 8:**
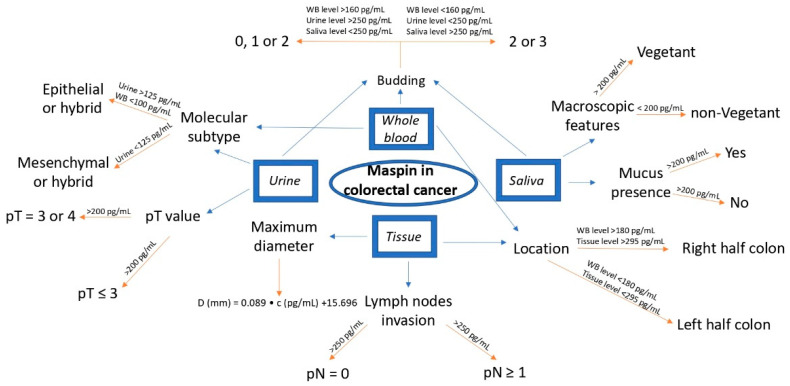
Workflow used for anticipation of pathological features in colorectal adenocarcinoma patients.

To summarize the discussions, a workflow was drawn, for the application of the findings (Figure 8):

## 5. Conclusions

Maspin is a biomarker with extremely useful possible medical applications. The correlations made in this paper will help with the fast diagnostic of colorectal adenocarci-nomas, after which it will be tested in a significant number of patients. At the moment, when the specimen is sent to the pathology department, it takes several days for processing and even more days if immunohistochemistry is necessary. For example, in some cases, budding is hard to appreciate and it is necessary to use maspin as an immunohistochemistry marker. The utilization of workflow may facilitate a faster diagnostic, and will provide to the surgeon information needed for the personalized protocol of treatment.

## Figures and Tables

**Table 1:** Budding and number of patients by the concentration of maspin in whole blood.

Maspin Concentration < 160 pg/mL			Maspin Concentration > 160 pg/mL		
**No = 14**			**No = 15**		
Budding	No	%	Budding	No	%
0	0	0.00%	0	2	13.33%
1	2	14.29%	1	5	33.33%
2	6	42.86%	2	6	40.00%
3	6	42.86%	3	2	13.33%
0 and 1	2	14.29%	0 and 1	7	46.67%
2 and 3	12	85.71%	2 and 3	8	53.33%

**Table 2:** The concentration of maspin in whole blood versus molecular subtype of colon cancer.

Whole Blood		Molecular Subtype	Whole Blood		Molecular Subtype
Maspin (pg/mL)	SD		Maspin (pg/mL)	SD	
4.56	0.06	Epithelial	189.25	0.03	Epithelial
38.59	0.02	-	191.78	0.03	Hybrid
51.68	0.03	Epithelial	195.6	0.04	Epithelial
53.14	0.02	-	198.68	0.03	Epithelial
59.08	0.02	Epithelial	201.9	0.05	Epithelial
69.08	0.03	Epithelial	201.9	0.03	Epithelial
69.08	0.05	Epithelial	274.3	0.03	Hybrid
100.7	0.04	Hybrid	286.65	0.02	Hybrid
119.68	0.04	Hybrid	330.93	0.04	Hybrid
130.87	0.02	Hybrid	360.72	0.05	Epithelial
136.41	0.03	Mesenchymal	366.35	0.04	Hybrid
147.76	0.04	-	424.67	0.03	Epithelial
150.91	0.05	Hybrid	436.87	0.03	Epithelial
151.3	0.05	-	454.63	0.04	Hybrid
165.84	0.02	Hybrid	454.9	0.02	Epithelial
182.36	0.04	Epithelial			

**Table 3:** Correlation of maspin concentration in whole blood and location of the tumor.

Location	Maspin (pg/mL)	Average Value of Maspin Concentration (pg/mL)	Maspin Concentration < 180 pg/mL				Maspin Concentration > 180 pg/mL			
			Number	%	Number	%	Number	%	Number	%
Ascending colon	130.87	261.51	1	16.67	2	22.22	5	83.33	7	77.78
	182.36									
	195.60									
	198.68									
	424.67									
	436.87									
	69.08	209.07	1	33.33			2	66.67		
Transverse colon	191.78									
	366.35									
Descending colon	4.56	21.58	2	100.00	13	59.09	0	0.00	9	40.91
	38.59									
Sigmoid colon	150.91	216.21	2	50.00			2	50.00		
	151.30									
	201.90									
	360.72									
Rectosigmoid colon	330.93	186.06	3	50.00			3	50.00		
	51.68									
	53.14									
	119.68									
	274.30									
	286.65									
Rectum	59.08	197.96	6	60.00			4	40.00		
	69.08									
	100.70									
	136.41									
	147.76									
	165.84									
	201.90									
	454.63									
	454.90									
	189.25									

**Table 4:** Correlation between the values of the concentration of maspin in tumoral tissue and the location of tumor.

Location			Maspin (pg/mL)	Average Value of Maspin Concentration (pg/mL)	Maspin Concentration < 180 pg/mL				Maspin Concentration > 180 pg/mL			
					Number	%	Number	%	Number	%	Number	%
Ascending colon			170.30	404.93	1	16.67	2	22.22	5	83.33	7	77.78
			295.64									
			369.47									
			500.98									
			537.08									
			556.10									
			100.11	263.34	1	33.33			2	66.67		
Transverse colon			296.78									
			393.14									
Descending colon			120.65	160.77	2	100.00	18	85.71	0	0.00	3	14.29
			200.89									
Sigmoid colon			153.20	214.50	4	100.00			0	0.00		
			223.75									
			237.32									
			243.74									
Rectosigmoid colon			239.25	310.59	4	80.00			1	20.00		
			145.61									
			274.49									
			291.59									
			602.02									
			131.47									
Rectum			217.99	312.88	8	80.00			2	20.00		
			219.88									
			222.45									
			222.46									
			252.50									
			280.58									
			301.86									
			973.14									
			125.06									

**Table 5:** Correlation of salivary maspin with budding.

Budding		0	1	2	3
Number for values of maspin concentration	<250 pg/mL	0	4	6	1
	>250 pg/mL	1	1	4	3
Total		1	5	10	4
Percents per values of maspin concentration	<250 pg/mL	0%	80%	60%	25%
	>250 pg/mL	100%	20%	40%	75%

**Table 6:** Correlation of salivary maspin concentration with the macroscopic vegetant feature.

Macroscopic Feature	Maspin Concentration < 200 pg/mL		Maspin Concentration > 200 pg/mL	
	No	%	No	%
Vegetant	2	25.00%	5	35.71%
Non-vegetant	6	75.00%	9	64.29%

**Table 7:** Correlation of urinary maspin concentration values with budding.

Values of maspin concentration in urine samples	Over 250 pg/mL	Budding	0	1	2	3
		Number	1	2	3	2
		Percent	12.5%	25%	37.5%	25%
	Under 250 pg/mL	Budding	0	1	2	3
		Number	0	1	7	5
		Percent	0%	7.69%	53.85%	38.46%

**Table 8:** Correlation of urinary maspin concentration with molecular subtype.

Value of maspin concentration in urine samples	>125 pg/mL	Molecular subtype	Epithelial	Hybrid	Mesenchymal
		Number	6	4	0
		%	60	40	0
	<125 pg/mL	Molecular subtype	Epithelial	Hybrid	Mesenchymal
		Number	3	6	1
		%	30	60	10

## Data Availability

All data used for this study are available in Supplementary Materials.

## Supplementary Materials

The following are available online at https://www.mdpi.com/article/10.3390/life13041060/s1, Table S1 Determination of the concentration of maspin in tumoral tissue, whole blood, saliva and urine using stochastic sensors, Table S2a Pathological features, Table S2b. Pathological features.
