# Supplementary material for: Correlation between Maspin Levels in Different Biological Samples and Pathologic Features in Colorectal Adenocarcinomas
Source: Life (Basel). 2023 Apr 20;13(4):1060. doi: 10.3390/life13041060 (PMC10143283; doi:10.3390/life13041060)
Supplement: Supplementary file 1 [file life-13-01060-s001.zip › life-2249971-supplementary.pdf]

Supplementary materials

Article type: Original article

Correlation between MASPIN levels in different biological samples and pathologic features in colorectal adenocarcinomas

Alexandru Adrian Bratei[1,2,3,4], Raluca-Ioana Stefan-van Staden[1,2]

[1] Faculty of Applied Chemistry and Material Sciences, University Politehnica of Bucharest, Bucharest, Romania
[2] Laboratory of Electrochemistry and PATLAB, National Institute of Research for Electrochemistry and Condensed Matter, 060021 Bucharest-6, Romania
[3] Department of Pathology, Emergency University Hospital, Bucharest, Romania
[4] Department of Pathology, University of Medicine and Pharmacy of Tirgu-Mures, 540139 Tirgu Mures, Romania

Corresponding Author:

Prof. dr. habil. R.I. Stefan-van Staden[1,2], Laboratory of Electrochemistry and PATLAB, National Institute of Research for Electrochemistry and Condensed Matter, 060021 Bucharest-6, Romania, email: ralucavanstaden@gmail.com

Table S1. Determination of the concentration of maspin in tumoral tissue, whole blood, saliva and urine using stochastic sensors.

Patient nr.	Tumoral tissue		Whole blood		Saliva		Urine	
	Maspin (pg/mL)	SD	Maspin (pg/mL)	SD	Maspin (pg/mL)	SD	Maspin (pg/mL)	SD
1	145.61	0.04	119.68	0.04	-	-	-	-
2	252.5	0.03	201.9	0.05	347.38	0.03	-	-
3	296.78	0.04	191.78	0.03	56.43	0.03	978	0.03
4	222.46	0.03	454.9	0.02	208.22	0.05	45.27	0.03
5	131.47	0.05	454.63	0.04	210.12	0.02	50.87	0.02
6	222.45	0.05	59.08	0.02	473.88	0.04	521.46	0.04
7	100.11	0.02	366.35	0.04	157.63	0.03	16.83	0.02
8	602.02	0.03	274.3	0.03	391.65	0.02	106.3	0.02
9	556.1	0.04	182.36	0.04	35.24	0.04	-	-
10	301.86	0.03	69.08	0.03	135.11	0.02	301.01	0.04
11	120.65	0.08	4.56	0.06	301.84	0.06	45.69	0.03
12	274.49	0.07	53.14	0.02	158.89	0.03	96.53	0.04
13	170.3	0.03	195.6	0.04	252.5	0.02	134.51	0.01
14	369.47	0.03	436.87	0.03	217.08	0.04	-	-
15	291.59	0.07	286.65	0.02	44.41	0.03	26.38	0.04
16	280.58	0.02	165.84	0.02	481.47	0.03	352.18	0.03
17	239.25	0.03	330.93	0.04	157.63	0.04	-	-
18	-	-	51.68	0.03	252.5	0.03	665.25	0.05
19	223.75	0.04	150.91	0.05	334.73	0.03	215.19	0.03
20	973.14	0.02	136.41	0.03	-	-	12.6	0.04
21	500.98	0.03	130.87	0.02	-	-	14.87	0.03
22	125.06	0.07	189.25	0.03	-	-	11.61	0.04
23	217.99	0.04	100.7	0.04	277.8	0.05	913.1	0.04
24	237.32	0.02	360.72	0.05	249.34	0.02	-	-
25	537.08	0.03	424.67	0.03	-	-	-	-
26	243.74	0.04	201.9	0.03	-	-	378.27	0.04
27	200.89	0.03	38.59	0.02	78.69	0.04	24.96	0.03
28	153.2	0.04	151.3	0.05	-	-	847.56	0.04
29	219.88	0.03	147.76	0.04	360.66	0.03	42.73	0.04
30	393.14	0.03	69.08	0.05	-	-	-	-

31	295.64	0.02	198.68	0.03	-	-	606.57	0.03

Table S2a. Pathological features.

Patient nr.	Type	Sex	Age	Exact Location	Macroscopic features	Microscopic type
1	colorectal cancer	Male	85	Rectosigmoid colon	Ulcero-infiltrative	Adenocarcinoma
2	colorectal cancer	Male	72	Rectum	Vegetant	Adenocarcinoma
3	colorectal cancer	Male	69	Transverse colon	Ulcero-infiltrative	Adenocarcinoma
4	colorectal cancer	Male	75	Rectum	Ulcero-infiltrative	Adenocarcinoma
5	colorectal cancer	Male	70	Rectum	Vegetant and ulcerated	Adenocarcinoma
6	colorectal cancer	Female	79	Rectum	Vegetant and ulcerated	Adenocarcinoma
7	colorectal cancer	Female	57	Transverse colon	Ulcero-infiltrative	Adenocarcinoma
8	colorectal cancer	Male	55	Rectosigmoid colon	Ulcero-infiltrative	Adenocarcinoma
9	colorectal cancer	Male	83	Ascending colon	Vegetant and ulcerated	Adenocarcinoma
10	colorectal cancer	Male	67	Rectum	Ulcero-infiltrative	Adenocarcinoma
11	colorectal cancer	Male	70	Descending colon	Ulcero-infiltrative	Adenocarcinoma
12	colorectal cancer	Male	72	Rectosigmoid colon	Vegetant and ulcerated	Adenocarcinoma
13	colorectal cancer	Female	73	Ascending colon	Vegetant and ulcerated	Adenocarcinoma
14	colorectal cancer	Male	81	Ascending colon	-	Adenocarcinoma
15	colorectal cancer	Male	80	Rectosigmoid colon	Ulcero-infiltrative	Adenocarcinoma
16	sigmoid cancer	Female	47	Rectum	Ulcero-infiltrative	Adenocarcinoma
17	sigmoid cancer	Male	79	Rectosigmoid and anal canal	Ulcero-infiltrative	Adenocarcinoma
18	Rectosigmoid cancer	Male	45	Rectosigmoid colon	Ulcero-infiltrative	Adenocarcinoma
19	sigmoid cancer	Male	63	Sigmoid colon	Vegetant	Adenocarcinoma
20	Rectal cancer	Male	75	Rectum	Vegetant	Adenocarcinoma
21	ascending colon cancer	Male	76	Ascending colon	Vegetant and ulcerated	Adenocarcinoma
22	Rectal cancer	Female	63	Rx Rectum	Ulcero-infiltrative	Adenocarcinoma
23	Rectal cancer	Female	56	Rectum	-	Adenocarcinoma
24	sigmoid cancer	Male	78	Sigmoid colon	Ulcero-infiltrative	Adenocarcinoma
25	ascending colon cancer	Female	72	Ascending colon	Ulcero-infiltrative	Medullary adenocarcinoma
26	sigmoid cancer	Male	75	Sigmoid colon	Ulcero-infiltrative	Adenocarcinoma
27	descending colon cancer	Female	64	Descending colon	Ulcero-infiltrative	Adenocarcinoma
28	sigmoid cancer	Female	86	Sigmoid colon	Ulcero-infiltrative	Adenocarcinoma

29	rectal cancer	Male	68	Rectum	Ulcero-infiltrative	Adenocarcinoma
30	transverse colon cancer	Male	70	Transverse colon	Ulcero-infiltrative	Adenocarcinoma
31	cecum carcinoma	Male	76	Ascending colon	Vegetant	Medullary adenocarcinoma

Table S2b. Pathological features.

Patient nr.	Adenocarcinoma Grading	Maximum diameter (mm)	Maximum depth (mm)	Mucinous compound	Molecular subtype	Survival	pT	pN	Budding	Stroma
1	G2	60	22	No	Hybrid	Dead	3	0	1	fibrous-inflammatory
2	G2	40	21	No	Epithelial	Alive	2	0	0	-
3	G2	40		No	Hybrid	Dead	4	0	2	fibrous-inflammatory
4	G2	50	25	Yes	Epithelial	Alive	3	1	2	fibrous-inflammatory
5	G2	60	16	No	Hybrid	Alive	3	1	2	fibrous-inflammatory
6	G2	40	25	Yes	Epithelial	Alive	4	1	3	fibrous-inflammatory
7	G2	22	12	No	Hybrid	Alive	3	1	2	fibrous-inflammatory
8	G2	60	-	No	Hybrid	Dead	4	1	2	fibrous-inflammatory
9	G2	80	-	No	Epithelial	Alive	3	0	1	fibrous-inflammatory
10	G2	70	13	No	Epithelial	Alive	3	0	1	fibrous-inflammatory
11	G2	30	9	No	Epithelial	Alive	3	1	2	fibrous-inflammatory
12	G2	23	10	No	-	Alive	3	0	2	fibrous-inflammatory
13	G2	40	12	Yes	Epithelial	Alive	4	1	3	-
14	G2	-	-	No	Epithelial	Alive	3	2	2	fibrous-inflammatory
15	G2	45	10	No	Hybrid	Alive	3	0	1	fibrous-inflammatory
16	G2	30	12	Yes	Hybrid	Alive	3	2	1	-
17	G2	40	12	No	Hybrid	Alive	4	1	1	inflammatory
18	G2	30	7	No	Epithelial	Alive	4	0	2	fibrous-inflammatory
19	G2	60	20	Yes	Hybrid	Alive	4	1	3	inflammatory
20	G2	38	-	No	Mesenchymal	Alive	3	0	3	fibrous-inflammatory
21	G2	65	13	Yes	Hybrid	Alive	3	0	3	fibrous-inflammatory
22	G2	20	-	No	Epithelial	Alive	2	0	-	-
23	G3	30	12	No	Hybrid	Alive	4	0	-	fibrous-inflammatory
24	G2	30	13	Yes	Epithelial	Alive	4	0	-	-
25	G1	65	15	No	Epithelial	Alive	2	0	1	-
26	G2	30	-	No	Epithelial	Alive	4	1	3	fibrous-inflammatory

27	G2	30	15	No	-	Alive	4	2	3	fibrous-inflammatory
28	G2	50	30	No	-	Alive	4	0	2	-
29	G2	20	15	No	-	Dead	4	1	2	-
30	G2	45	17	No	Epithelial	Alive	4	1	3	-
31	G1	95	77	No	Epithelial	Alive	4	0	0	fibrous-inflammatory
